# Thermoelectric Characteristics of A Single-Crystalline Topological Insulator Bi_2_Se_3_ Nanowire

**DOI:** 10.3390/nano11030819

**Published:** 2021-03-23

**Authors:** Dedi, Ping-Chung Lee, Pai-Chun Wei, Yang-Yuan Chen

**Affiliations:** 1 Research Center for Electronics and Telecommunications (P2ET), Indonesian Institute of Sciences (LIPI), Bandung 40135, Indonesia; 2 Institute of Physics, Academia Sinica, Taipei 11529, Taiwan; plexlee@gmail.com (P.-C.L.); cheny2@phys.sinica.edu.tw (Y.-Y.C.); 3 Center for Condensed Matter Sciences and Center of Atomic Initiatives for New Materials, National Taiwan University, Taipei 10617, Taiwan; pcwei68@gmail.com

**Keywords:** thermoelectric, bismuth selenide, nanowire

## Abstract

The discovery of topological insulators (TIs) has motivated detailed studies on their physical properties, especially on their novel surface states via strong spin–orbit interactions. However, surface-state-related thermoelectric properties are rarely reported, likely because of the involvement of their bulk-dominating contribution. In this work, we report thermoelectric studies on a TI bismuth selenide (Bi_2_Se_3_) nanowire (NW) that exhibit a larger surface/volume ratio. Uniform single-crystalline TI Bi_2_Se_3_ NWs were successfully synthesized using a stress-induced growth method. To achieve the study of the thermoelectric properties of a nanowire (NW), including electrical conductivity (σ), Seebeck coefficient (S), and thermal conductivity (κ), a special platform for simultaneously performing all measurements on a single wire was designed. The properties of σ, S, and κ of a 200 nm NW that was well precharacterized using transmission electron microscope (TEM) measurements were determined using the four-probe method, the two-probe EMF across ∇T measurement, and the 3ω technique, respectively. The integrated TE properties represented by the figure of merit ZT (S^2^σT/κ) were found to be in good agreement with a theoretical study of Bi_2_Se_3_ NW.

## 1. Introduction

The study of nanoengineered thermoelectric materials used for converting waste heat into electricity has become a compelling research topic [1,2,3,4,5]. The thermoelectric (TE) generator and the TE sensor are devices that can harvest renewable energy for power generation and thermal sensing, respectively [6,7,8,9,10,11]. The efficiency of TE materials is determined by the dimensionless figure of merit *ZT*, which is defined as *S^2^σT/(κ_e_ + κ_l_)*, where *S* is the TE power or Seebeck coefficient, *σ* represents the electrical conductivity, *κ_e_* is the electronic thermal conductivity, and *κ_l_* is the lattice thermal conductivity. The quantity *S^2^σ* is defined as the power factor (PF). The Weidmann–Franz law restricts the ratio σ/κ in a bulk TE compound. Furthermore, a sharply peaked density of states (DOS) favors a large S, while the density of states in bulk materials is a smoothly variable function. As dimensionality is reduced from three to one, the electronic DOS at the energy-band edges is significantly increased, increasing the TE PF (*S^2^σ*) and yielding an improved ZT [12,13]. Then, according to Dresselhaus et al., 1D nanowires can boost thermoelectric performance [13,14]. A Bi nanowire would also have a reduced *κ* due to phonon scattering off the sidewalls, which helps increase ZT, based on a previous study [13,15].

Slack et al. reported that semiconductors exhibiting narrow band gaps and high mobility carriers are optimal TE materials [16]. Bi_2_Se_3_ is a V–VI topological-insulator material that exhibits a narrow band gap of approximately 0.3 eV and crystallizes in a rhombohedral structure belonging to the tetradymite space group $D_{3d}^{5}$ (R-3m) [17,18,19]. This material demonstrates potential for application in optical recording systems [20], photo-electrochemical devices [21], and TE devices [17,18]. In recent years, bismuth chalcogenides have attracted substantial research interest because of their superior TE properties of high *ZT* at room temperature [22]. Diverse synthesis techniques have been developed to synthesize various nanostructures of Bi_2_Se_3_, such as microwave heating [23,24], a single-source precursor method [25,26,27], solvothermal method [28,29,30], the metal–organic chemical vapor deposition method [31,32], and mechanical exfoliation [33,34,35], whereas the commonly used synthesis method for producing Bi_2_Se_3_ bulk single-crystalline material is based on the Bridgman technique [36,37,38,39,40,41]. However, only a few studies have been reported on growing Bi_2_Se_3_ NWs and characterizing their TE properties.

## 2. Materials and Methods

We previously synthesized PbTe NWs from a PbTe thin film (TF) on a SiO_2_/Si substrate using a stress-induced method [42,43] based on a mechanism in which mismatched thermal expansion between a substrate and deposited film drove the mass flow along grain boundaries at thermal annealing temperatures. This innovative NW-growth method (which does not involve conventional templates, catalysts, or starting materials) enables us to control growth conditions for growing different diameters, shapes, and aspect ratios of single-crystalline NWs [44,45], thus enabling exploration of any possible novel TE property of Bi_2_Se_3_ NWs.

The starting Bi_2_Se_3_ crystalline ingot was synthesized from Bi and Se source materials using the Bridgman method. Bi (99.999%, −200 mesh, Alfa Aesar, Lancashire, UK) and Se (99.999%, −200 mesh, Alfa Aesar, Lancashire, UK) powders were first mixed at a 2:3 ratio and then melted at 800 °C for 4 h in a vacuumed quartz tube at a pressure less than 5 × 10^−6^ torr. The molten compound was slowly cooled in the furnace to room temperature. Subsequently, a pellet specimen cut from the compound served as the target for pulsed laser deposition (PLD). Single-crystal SiO_2_/Si (100) wafers (E-light Tech. Inc. Taipei, Taiwan; SiO_2_ thickness = 1µm; diameter = 100 ± 0.5 mm) with double-side polishing were cut into 1.5 × 1.5 cm^2^ squares for substrates. All substrates were cleaned using acetone, isopropyl alcohol, and deionized water in an ultrasonic bath for 10 min before being dried with an N_2_ stream. The Bi_2_Se_3_ films were fabricated using an ArF excimer laser (Lambda Physik LPXPro 210, Santa Clara, California, USA) and deposited onto substrates in a vacuum system with a base pressure of 5.0 × 10^−7^ torr. The Bi_2_Se_3_ TFs were grown at a deposition rate of 0.3 Å/s, and the excimer laser was applied at 140 mJ (frequency = 10 Hz) for 15 min at room temperature. The substrate was rotated at approximately 10 rpm, and the film thickness was 30 nm. The films were sealed in a vacuumed quartz tube at less than 5 × 10^−6^ torr for annealing at 450 °C for 5 d, followed by cooling the furnace to room temperature. During the annealing process, Bi_2_Se_3_ NWs grew from the film via the different thermal expansion coefficients of the Bi_2_Se_3_ film (19 × 10^−6^/°C) [46] and the SiO_2_/Si substrate (0.5 × 10^−6^/°C)/(2.4 × 10^−6^/°C) [42,43,44,45].

## 3. Results and Discussion

### 3.1. Characterization of Materials

**Figure 1:**
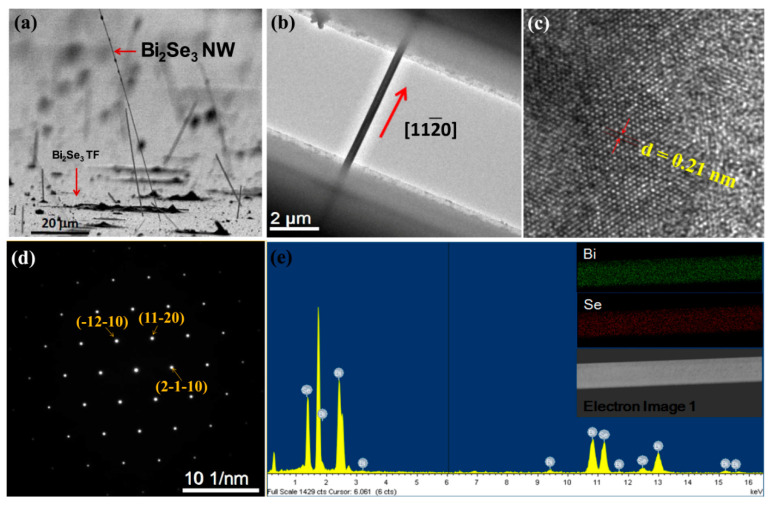
(**a**) SEM image (top view) indicating that all Bi_2_Se_3_ NWs grew several micrometers in length from the surface of the Bi_2_Se_3_ TF; (**b**) TEM images of a single-crystal Bi_2_Se_3_ NW suspended on a Si_3_N_4_ microchip following electrode formation by using a FIB that served as the TEM holder; (**c**) HR-TEM image of the NW shown in (**b**), where the distance between crystal faces is 0.21 nm; (**d**) the SAED pattern (at the [0001] zone axis), confirming that the single-crystalline NWs grew in the [11,12,13,14,15,16,17,18,19,20] direction; and (**e**) EDS spectrum of a Bi_2_Se_3_ NW (the inset shows the EDS mapping image of a NW).

Field emission scanning electron microscopy (Hitachi Co., S-4800, Tokyo, Japan) images of the Bi_2_Se_3_ NWs (Figure 1a) showed that the NWs exhibited diameters ranging from 50 to 500 nm and lengths up to 100 µm. Straight and uniform Bi_2_Se_3_ NWs of high aspect ratio grew on the substrate after annealing. A tungsten needle (*d* = 100 nm) and a binocular optical microscope were used to extract a single-crystal NW from the Bi_2_Se_3_ film; the NW was then suspended on a Si_3_N_4_ microchip by using electrodes (Figure 1b) and employed in structural analyses and thermoelectricity measurements. A transmission electron microscope (TEM; JEOL JEM-2100 at 200 kV, Tokyo, Japan) was used to examine the crystalline structure of the Bi_2_Se_3_ NW (Figure 1c,d).

The electrical resistivity *ρ* and Seebeck coefficient *S* of the Bi_2_Se_3_ bulk were measured simultaneously using commercial equipment (ZEM-3, ULVAC-RIKO, Chigasaki, Kanagawa, Japan) in a He atmosphere from 300 to 540 K. The thermal conductivity *κ* of the Bi_2_Se_3_ bulk was calculated using the equation *κ* = *D.C_p_.d*, where *D* is the thermal diffusivity, *C_p_* is the specific heat, and *d* is the density of the sample. The thermal diffusivity *D* was examined via a laser-flash apparatus (NETZSCH, LFA 457, Selb, Germany), and the density d was obtained using the Archimedes method (presented in Figure S1, electronic supporting information (ESI)). The Seebeck coefficient *S* and the electrical resistivity *ρ* of an NW were measured using a conventional steady-state method in an Oxford cryostat. The NW thermal conductivity was measured using the 3ω method.

The TEM image (TEM; JEOL JEM-2100 at 200 kV, Tokyo, Japan) in Figure 1c reveals that the ordered hexagonal structure exhibiting lattice fringes of 0.21 nm gaps between the [11,12,13,14,15,16,17,18,19,20] planes were consistent with those of other lattice spacing measurements of Bi_2_Se_3_ NWs [47]. A corresponding selected area electron diffraction (SAED) pattern (Figure 1d) reveals that the Bi_2_Se_3_ NWs were high-quality single crystals that exhibited growth along the [11,12,13,14,15,16,17,18,19,20] direction. The chemical composition of the Bi_2_Se_3_ NW was examined using energy dispersive X-ray spectroscopy (EDS, JEOL, Tokyo, Japan); the EDS mapping shown in the inset of Figure 1e indicates the uniform spatial distribution of Bi and Se elements throughout the NW.

**Figure 2:**
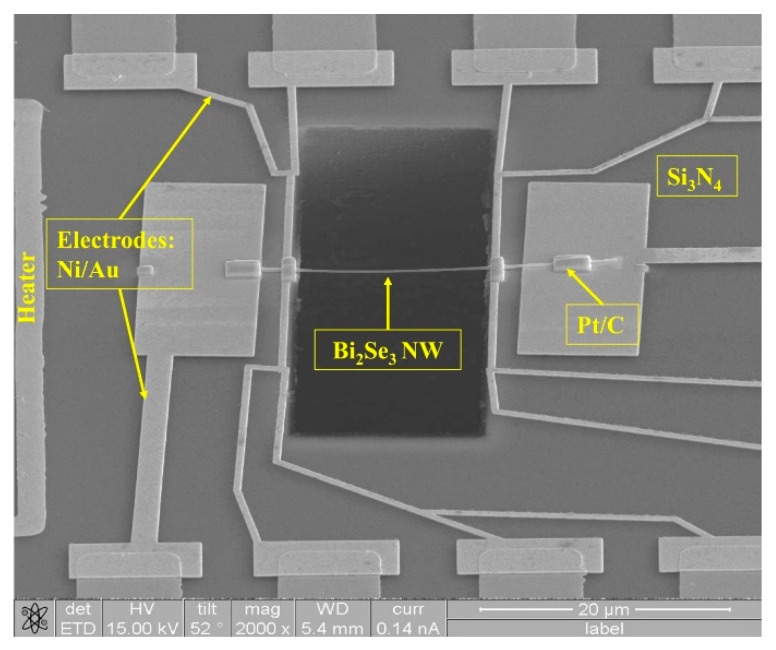
The SEM image of a single-crystal Bi_2_Se_3_ NW (diameter = 200 nm) placed on a Si_3_N_4_ microchip following electrode formation by using a FIB.

Figure 2 shows a SEM image of a Bi_2_Se_3_ NW (d = 200 nm) suspended on a Si_3_N_4_ microchip between 10 nm Ni/50 nm Au electrodes and a Pt/C electrical contact deposited by a focus ion beam (FIB). The sample used in this study showed nearly an ohmic contact. Subsequently, the microchip was used to determine the values of electrical resistivity *ρ* and *S* via four-point measurements.

### 3.2. Characterization of Thermoelectric Properties 

**Figure 3:**
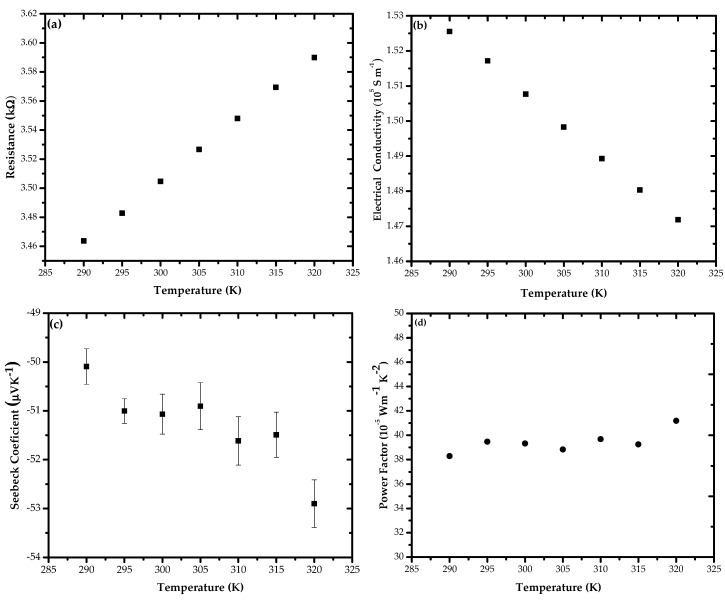
Temperature dependence regarding the (**a**) electrical resistance, (**b**) electrical conductivity, (**c**) TE power (*S*) and (**d**) power factor of the single-crystal Bi_2_S_3_ NW.

Figure 3a shows the temperature dependence of the electrical resistance at 290–320 K for a 200 nm Bi_2_Se_3_ NW that exhibited weak metallic conductivities. The *σ* of the NW at near room temperature was 1.50767 × 10^5^ S m^−1^ (Figure 3b), which was approximately 55% lower than that of the Bi_2_Se_3_ bulk single-crystal (2.7550 × 10^5^ S m^−1^) [41] (Table 1). Furthermore, the surface scattering of charge carriers typically yields a reduced *σ* value [48]. However, the *σ* values of the Bi_2_Se_3_ NW at room temperature was higher than those reported in previous studies on the Bi_2_Se_3_ bulk [23,24,25,26,28,29,32], and even higher than that of the Bi_2_Se_3_ single-crystal made by Hor et al. [39], probably the result of the increased contribution of conduction surfaces up to 73% of the total electrical conduction upon decreasing the NW dimension via the high surface-to-volume ratio s/v ~ 2 nm^−1^ of the nanowires. A previous report [19] of electrical transport experiments on Bi_2_Te_3_ and Bi_2_Se_2_Te nanowires in the range of 200–300 nm in diameter revealed that the two-dimensional TI surface channels contribute up to 30–70% of the total electrical conduction at surface-to-volume ratios of s/v = 2−5 × 10^−2^ nm^−1^.

The *S* values with the negative sign obtained for the Bi_2_Se_3_ NW (Figure 3c) show that the Bi_2_Se_3_ NW is an n-type semiconductor, because electrons have much higher mobility than holes and dominate the transport [49,50]. This is reasonable because undoped Bi_2_Se_3_ is strongly n-type [51]. Furthermore, the room temperature *S* values for the *n*-type Bi_2_Se_3_ NW was −51 μVK^−1^ for the 200 nm NW, the value was comparable to those typically reported by Greenaway and Harbeke for this material (i.e., in the −55 to −73 μVK^−1^ range) [52], indicating that the Fermi level lay well inside the conduction band. The magnitude of the *S* smoothly increased as the temperature increased. This behavior was consistent with that expected of a metallically doped material. The magnitude of *S* for the NW tends to be zero when the temperature is decreased because *S* represents the entropy per electric charge and must decrease to zero at 0 K [15]. Figure 3d indicates the temperature dependence of the *PF* of Bi_2_Se_3_ NW, indicating that the *PF* increased as temperature increased; this can be attributed to the increase in the *S* with the temperature of the Bi_2_Se_3_ NW. The *PF* value of the 200 nm Bi_2_Se_3_ NW at room temperature was 39.32 × 10^−5^ Wm^−1^K^−2^, which was higher than the *PF* of the Bi_2_Se_3_ bulk nanostructures [21,22,23,24,26,27,31]. The enhanced *PF* value is likely a result of enhanced electronic transport of the NW.

**Figure 4:**
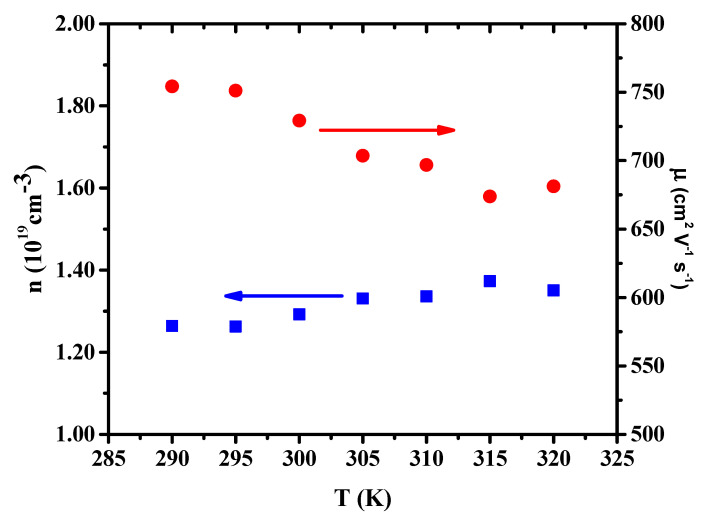
Temperature dependence of carrier concentration and carrier mobility for Bi_2_Se_3_ NW.

For semiclassical transport (metals or degenerate semiconductors) the carrier-density dependence of the thermopower is described by the Mott relation [53,54,55]:

(1) $$S=\frac{8\pi^{2}k_{B}^{2}T}{3qh^{2}}m^{*}{\left(\frac{\pi }{3n}\right)}^{\frac{2}{3}}$$

where *k*_B_ is Boltzmann’s constant, *q* is the electron charge, *h* is Planck’s constant, *T* is the measurement temperature, m* is the effective mass of the carrier (m* = 0.14 *m_o_* in Bi_2_Se_3_) [56] and *m_o_* is the electron mass. This formula is valid for assessing metals or degenerate semiconductors that exhibit an *n* value in the range of 10^18^ to 10^20^ cm^−3^ [57,58]. The value of *n* is in the range of 1.26–1.35 × 10^19^ cm^−3^ at 290–320 K for the 200 nm NW (Figure 4), indicating that the NW is a degenerate semiconductor. This value is close to that calculated by Boechko et al. for n-type single crystals of a Bi_2_Se_3_ single crystal (1–4 × 10^19^ cm^−3^) [59]. The value of *n* increased as the temperature increased, indicating the intrinsic condition [60], with the number of thermally generated carriers exceeding the number of donor carriers. The intrinsic carrier concentration in a material *n_i_* is generally much smaller than the dopant carrier concentration at room temperature, but *n_i_* (*=n·p*) has a very strong temperature dependence:

(2) $$\;\;n_{i}\propto T^{1.5}e^{-\frac{E_{g0}}{2kT}}$$

where *E_g_*_0_ is the energy band gap at *T* = 0 *K* [60].

Figure 4 also depicts the calculated *T* dependences of carrier mobility *μ* for the Bi_2_Se_3_ NW. The *μ* value is obtained using the following equation:

(3) $$\mu =\frac{1}{\rho nq}=\;\frac{\sigma }{nq}$$

Our calculated values of *μ* at 290–320 K were 754–681 cm^2^ V^−1^ s^−1^ for the 200 nm NW; these values were much smaller than those of the Bi_2_Se_3_ bulk (approximately 920–1060 cm^2^ V^−1^ s^−1^) [41], but higher than those reported by Le et al. [60] for Bi_2_Se_3_ TFs (7.2 ± 0.2 to 98.4 ± 0.5 cm^2^ V^−1^ s^−1^). The *μ* values decreased as temperature increased because of the phonon concentration increase that caused increased scattering. Thus, lattice scattering reduced the carrier mobility at higher temperature. The mobility of a semiconductor depends on the impurity concentrations (including donor and acceptor concentrations), defect concentration, temperature, and electron and hole concentrations.

The primary factor involved in determining *μ* in the semiconductor is the scattering mechanism through the relation $\;\mu_{j}\propto T^{\alpha }$ [61]. Conduction carriers are scattered by acoustic phonons $\;\mu_{l}$ when $\;\alpha =-\frac{3}{2}$, whereas they are scattered by ionized impurities $\;\mu_{i}$ when $\;\alpha =\frac{3}{2}$. The *μ* values of the 200 nm Bi_2_Se_3_ NW continually decreased as the *T* increased, indicating that phonon scattering was dominant throughout the whole temperature range.

**Figure 5:**
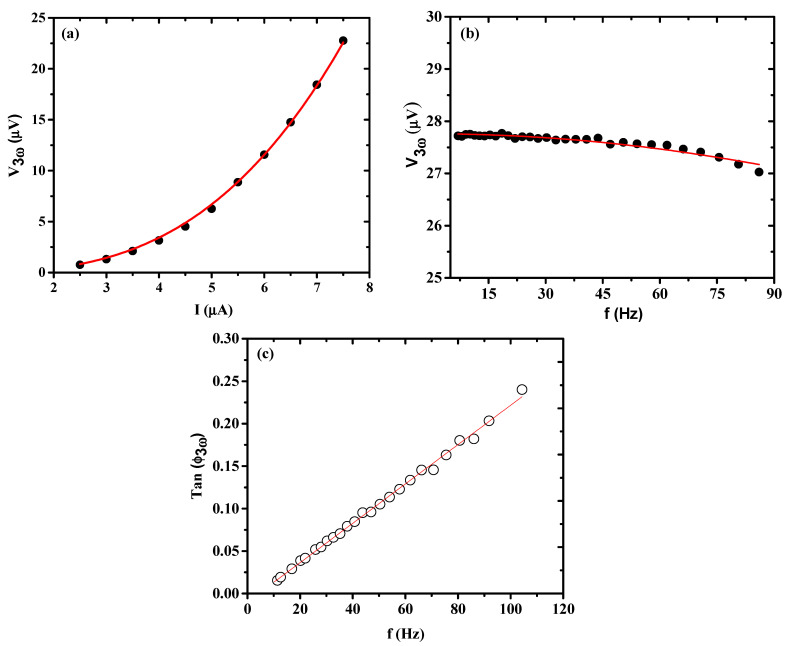
(**a**) The third harmonic voltage signal V3ω as function of the extraction current amplitude I_o_. The solid line shows the cubic relationship of V3ω and I_o_. (**b**) Frequency dependence of V3ω. The solid line is the predicted relation $V3\omega \;\alpha \;\;1/\sqrt{1+{\left(2\omega \gamma \right)}^{2}}$. (**c**) The frequency dependence of the phase angle of V3ω at 300 K of Bi_2_Se_3_ NW; d = 200 nm.

The thermal conductivity of Bi_2_Se_3_ NW was measured by the self-heating 3ω method in the temperature range of 290–320 K. The 3ω signal can be expressed as [62,63]:

(4) $$V_{3\omega }=\frac{4I_{0}^{3}{LRR}^{\prime }}{\pi^{4}\kappa S\sqrt{1\;+\;{\left(2\omega \gamma \right)}^{2}}}$$

where *I* and *ω* are the amplitude and frequency of the alternating current applied on the nanowire, respectively; *R* and *R′* are the resistance and derivative of resistance at the corresponding temperature, respectively; κ is the thermal conductivity; *S* is the NW cross-section area; and *γ* is the characteristic thermal time constant. Figure 5a shows the current dependence of V_3ω_ at 300 K, demonstrating an I_0_ dependence in an intermediate current range; one can see that V_3ω_(I_0_) followed the I_0_^3^ dependence well, in agreement with Equation (4). Figure 5b,c show the frequency dependencies of the amplitude and the phase angle of *V*_3ω_ at 300 K, respectively, compared with the predicted functional forms (the solid lines). The fitting parameters for Figure 5a,b are shown in Tables S1 and S2, respectively.

**Figure 6:**
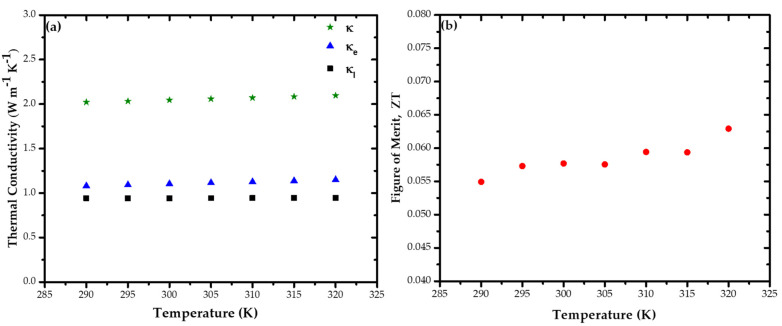
(**a**) Thermal conductivity κ, and (**b**) figure of merit *ZT* values of Bi_2_Se_3_ NW in the temperature range of 290 K to 320 K.

By fitting the data in Figure 5a to equation $\;V_{3\omega }=\frac{4I_{0}^{3}{LRR}^{\prime }}{\pi^{4}\kappa S}$ (ωγ→ 0), we obtained the thermal conductivity *κ,* and the thermal time constant *γ* was ~2 ms at 300 K, comparable to a simple theoretical calculation of *κ* based on the Callaway model for nanostructured Bi_2_Se_3_ made by Li et al. [64,65]. It is known that phonon-boundary scattering can suppress the thermal conductivity in nanowires [66,67]. However, the data on a Bi_2_Se_3_ NW with d = 200 nm from the experimental *κ* values (*κ* = 2.02 to 2.09 W m^−1^ K^−1^ at 290–320 K) as plotted in Figure 6a are in reasonable agreement with the Callaway model between the *κ* values of 300 nm (*κ* > 2 W m^−1^ K^−1^) and 100 nm (*κ* < 2 W m^−1^ K^−1^) [64]. The measured thermal conductivity, given by the *κ* value (*κ* is measured perpendicular to c plane) at T = 300 K of the NW (2.05 W m^−1^ K^−1^) was ~33% lower than those for Bi_2_Se_3_ bulk single-crystal (2.96 W m^−1^ K^−1^ or 3.1 W m^−1^ K^−1^ in Table 1) [39,41].

The electronic thermal conductivity *κ_e_* values of the Bi_2_Se_3_ NWs here were calculated according to the Wiedemann–Franz law:

*κ*_*e*_ = *LσT* (5)

where *L* is the Lorenz number (2.44 × 10^−8^ W Ω K^−2^). When subtracting the *κ_e_* values from the measured thermal conductivity, one obtains the phonon (or lattice) part of the thermal conductivity as *κ_l_*_=_ *κ-κ_e_*. The temperature-dependent data for *κ_l_* thus obtained are shown in Figure 6a. The obtained for *κ_l_* and *κ*_e_ at 300 K for NWs were 29% and 37% lower than that of the Bi_2_Se_3_ bulk single-crystal (*κ_l_* = 1.33 W m^−1^ K^−1^ and *κ_e_* = 1.77 W m^−1^ K^−1^) [41]. The thermal conductivity was dominated by the electronic contribution in the 290–320 K range, although Birkholz and Rosi both reported a value for *κ_l_* of Bi_2_Se_3_ bulk of between 2.0 and 2.4 W m^−1^ K^−1^ [51]. It was shown that the large surface-to-volume ratio s/v of nanowires could enhance phonon surface scattering and decrease *κ_l_*.

Additionally, for this nanowire, *ZT* calculated from the obtained *S*, σ, and *κ* was approximately 0.06 at 300 K (Figure 6b). However, the *ZT* values of this nanowire were still higher than those of Bi_2_Se_3_ bulk used to construct nanostructures at 290–320 K, as reported previously [26,28,29]. Table 1 shows a summary of the transport properties of single-crystalline Bi_2_Se_3_ NW, compared with those reported Bi_2_Se_3_ bulk at room temperature. Our *S, σ*, and *κ* results of Bi_2_Se_3_ NW *d* = 200 nm were in reasonable agreement with a theoretical study [19]. This agreement indicates the high-quality crystallinity of the Bi_2_Se_3_ NWs grown by the stress-induced method.

## 4. Conclusions

The stress-induced method was applied to grow single-crystalline Bi_2_Se_3_ nanowires (NWs) from a Bi_2_Se_3_ TF on a SiO_2_/Si substrate, offering an alternative technique for Bi_2_Se_3_ NWs synthesis without a catalyst. This technique had not been previously applied to Bi_2_Se_3_ alloys. In this work, at room temperature, the Bi_2_Se_3_ nanowire (NW) (d = 200 nm) exhibited a *PF* of approximately 39.32 × 10^−5^ Wm^−1^K^−2^, which was higher than that reported for Bi_2_Se_3_ bulk nanostructures; this discrepancy was mainly attributed to the electron-transport contribution of this NW. The measured thermal conductivity *κ* value of a NW was 2.05 W m^−1^ K^−1^, which was 31−34% lower than those for a Bi_2_Se_3_ bulk single crystal [39,41] because of the electron-scattering contribution. The figure of merit *ZT* value of Bi_2_Se_3_ NW rose up to approximately 0.06 at room temperature, in agreement with a theoretical study of the thermoelectric properties on a topological insulator of Bi_2_Se_3_ NWs [19]. Our results indicated that NWs grown using the stress-induced method yield high-quality single crystals.

## Figures and Tables

**Table 1:** The transport properties of Bi_2_Se_3_ nanowire and bulk at room temperature.

Sample *	S [µV K^−1^]	Σ [S m^−1^]	PF [10^−5^ W m^−1^K^−2^]	κ W m^−1^K^−1^	ZT	Ref.
Bi_2_Se_3_	−53	38678	10.70	0.78	0.04	[26]
Bi_2_Se_3_	−115	212	2.80	0.75	0.01	[28]
Bi_2_Se_2.83_	−60	25000	9	0.55	0.05	[29]
Bi_2_Se_3_ Nanowire SC (φ = 200 nm)	−51	150767	39.32	2.05	0.06	Our Work
Bi_2_Se_3_ Bulk SC	−62.10	259998	100	1.55	0.19	Our Work
Bi_2_Se_3_ Bulk SC	−190	47619	172	2.96	0.17	[39]
Bi_2_Se_3_ Bulk SC	−59	275500	95.90	3.1	0.09	[41]

* SC = single-crystalline.

## Data Availability

The data presented in this study are available on request from the corresponding author.

## Supplementary Materials

The following are available online at https://www.mdpi.com/2079-4991/11/3/819/s1, Figure S1: (a) Image of Bi_2_Se_3_ single-crystalline grown by the Bridgman method. (b) The measured thermoelectric properties were −62.10 μ VK^−1^, 259998 S m^−1^, 1.55 W m^−1^ K^−1^, and 0.19 for the Seebeck coefficient (S), electrical conductivity (σ), thermal conductivity (κ) and figure of merit *ZT*, respectively, at room temperature, as shown in Table 1, Table S1: The fitting parameters of the third harmonic voltage signal V3ω as function of the extraction current amplitude Io for Figure 5a, Table S2: The fitting parameters of frequency dependence of V3ω for Figure 5b.
