# Supplementary material for: Thermoelectric Characteristics of A Single-Crystalline Topological Insulator Bi2Se3 Nanowire
Source: Nanomaterials (Basel). 2021 Mar 23;11(3):819. doi: 10.3390/nano11030819 (PMC8005119; doi:10.3390/nano11030819)
Supplement: Supplementary file 1 [file nanomaterials-11-00819-s001.pdf]

nanomaterials

Supplementary Materials

Thermoelectric Characteristics of A Single-Crystalline Topological Insulator Bi_2Se_3 Nanowire

Dedi [1,3,*], Ping-Chung Lee [3], Pai-Chun Wei [2] and Yang-Yuan Chen [3]

1 Research Center for Electronics and Telecommunications (P2ET), Indonesian Institute of Sciences (LIPI), Bandung 40135, Indonesia
2 Center for Condensed Matter Sciences and Center of Atomic Initiatives for New Materials, National Taiwan University, Taipei 10617 Taiwan; pcwei68@gmail.com
3 Institute of Physics, Academia Sinica, Taipei 11529, Taiwan; plexlee@gmail.com (P.-C.L.); cheny2@phys.sinica.edu.tw (Y.-Y.C.).
* Correspondence: dedi030@lipi.go.id; Tel.: +62-22-250-4660

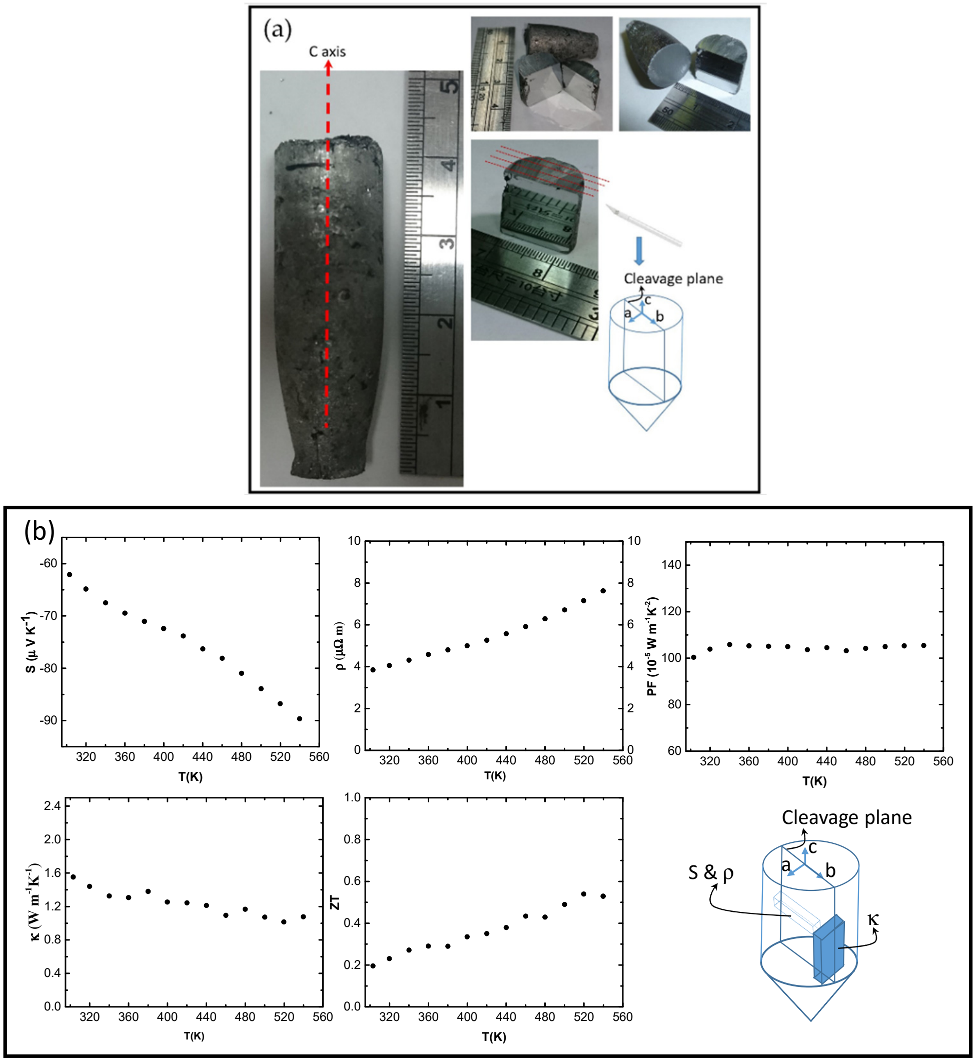

Figure S1. (**a**) Image of Bi_2Se_3 single crystalline was growth by Bridgman method and (**b**) Temperature dependent of thermoelectric properties of single crystalline Bi_2Se_3 bulk, which are −62.10 μ VK^{-1}, 259998 S m^{-1}, 1.55 W m^{-1}K^{-1}, and 0.19 for Seebeck coefficient (S), electrical conductivity (σ), thermal conductivity (κ) and figure of merit ZT, respectively at room temperature as shown in table 1.

Table S1. The fitting parameters of the third harmonic voltage signal V3ω as a function of the extraction current amplitude I_o for figure 5a.

Equation		$y = (K \cdot x^3)$	
Adj. R-Square	0.92439		
		Value	Standard Error
V3ω	K	5.35152×10^{-5}	3.21872×10^{-8}

Table S2. The fitting parameters of frequency dependence of V3ω for figure 5b.

Equation		$y = K \cdot (\frac{1}{\sqrt{1 + G \cdot x^2}} + 0.01)$	
Adj. R-Square	0.92439		
		Value	Standard Error
V3ω	K	2.74832×10^{-5}	1.14172×10^{-8}
V3ω	G	5.96359×10^{-6}	3.18552×10^{-7}

Copyright: © 2021 by the authors. Licensee MDPI, Basel, Switzerland. This article is an open access article distributed under the terms and conditions of the Creative Commons Attribution (CC BY) license (http://creativecommons.org/licenses/by/4.0/).
